# Report of two distinct ribotypes in ITS sequences of *Phalarisarundinacea* (Poaceae) in western Canada and Alaska

**DOI:** 10.3897/BDJ.11.e101257

**Published:** 2023-04-11

**Authors:** Diana M. Percy, Quentin C. B. Cronk

**Affiliations:** 1 Department of Botany, University of British Columbia, Vancouver, Canada Department of Botany, University of British Columbia Vancouver Canada; 2 Beaty Biodiversity Museum, University of British Columbia, Vancouver, Canada Beaty Biodiversity Museum, University of British Columbia Vancouver Canada

**Keywords:** internal transcribed spacer, invasive plant, *Phalaris*, reed canary grass

## Abstract

**Background:**

*Phalarisarundinacea* L. (reed canary grass) is a widely occurring grass throughout the Northern Hemisphere. In North America, it is thought to consist of introduced agricultural forms from Europe as well as native populations.

**New information:**

During a survey of *Phalarisarundinacea* in western Canada, we discovered two distinct ribotypes in the sequences of the internal transcribed spacer (ITS) of the nuclear ribosomal DNA: one full length (ITS-long) and one with a seven base pair deletion (ITS-short). In addition, ITS-long plants have fixed heterozygosity indicating possible polyploidy. Phylogenetic analysis reveals that ITS-short is a unique ribotype that characterises an intraspecific clade. We designed an efficient PCR-based assay that allows sizing of a 238/245 base pair fragment in a capillary sequencer. This approach provides a novel marker that could be useful in future surveys of *Phalarisarundinacea*.

## Introduction

**Figure 1.**
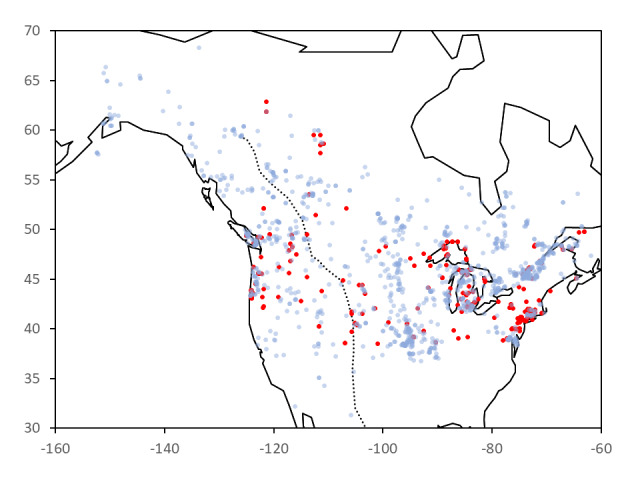
Map of North American *Phalarisarundinacea* herbarium specimens from the Global Biodiversity Information Facility (GBIF; accessed October 2021). Red = 1822-1940; blue = 1941-2018. The dotted line marks the boundary of the western cordilleras.

*Phalarisarundinacea* L., commonly called reed canary grass (RCG), is a Eurasian and North American perennial grass, with many uses in agriculture (Jakubowski et al. 2011) and biomass energy (Lewandowski et al. 2003). In North America, native populations are considered under threat from invasion and replacement by vigorous introduced genotypes of *P.arundinacea* that have now become a significant invader of wetland and riparian habitats in North America (Lavergne and Molofsky 2004) with considerable ecological impacts (Spyreas et al. 2010). The distribution of *Phalarisarundinacea* in North America, based on databased herbarium specimens, is shown in Fig. 1.

Molecular methods have often been used to distinguish populations of RCG, including isozymes (Gifford et al. 2002), AFLP (Casler et al. 2009), SSR (Jakubowski et al. 2013, Jakubowski et al. 2014, Kettenring et al. 2019), ISSR (Anderson et al. 2016), DartSeq (Noyszewski et al. 2019, Noyszewski et al. 2021) and ITS sequencing (Graper et al. 2021). However, there is still much uncertainty and, in some cases, disagreement, regarding the extent of distribution and location of present day native versus introduced RCG populations in North America (Jakubowski et al. 2013). The aim of this note is to detail an easily scored novel genetic marker that may be of use in future surveys of RCG.

## Sampling methods

### Study extent

**Sources of material - herbarium and field**

A total of 86 samples of *Phalarisarundinacea* were obtained from herbarium material and additional targeted sampling carried out for this study (Tables 1, 2, Suppl. materials 1, 3). Herbarium samples, from modern to 130 years old and in relatively good condition, were selected for sampling from the University of British Columbia Herbarium (UBC) and the Herbarium of the Bell Museum, University of Minnesota (MIN). Further dried leaf samples (used in a previous study; Kettenring et al. (2019)) were kindly provided by Professor Karen Mock of Utah State University. In addition, extensive field sampling was carried out in Elk Island National Park, Alberta, where park authorities were concerned about the ecologically harmful spread of, as well as appropriate control methods for, *Phalarisarundinacea*. Further recent samples were sourced from Greater Vancouver. Voucher specimens are deposited in UBC. As outgroups for the phylogenetic analyses, we used eight individuals obtained from herbarium samples of *P.aquatica* Guss., *P.canariensis* L., *P.caroliniana* Walter, *P.coerulescens* Desf. and *P.paradoxa* L. (Suppl. material 2).

### Step description

**DNA extraction, PCR and sequencing**

Dried leaf material was ground to a slurry in liquid nitrogen and the DNA extracted using a modified CTAB method (Doyle and Doyle 1987). Full length PCR (ITS1-5.8S-ITS2) was performed using primers ITS-A (forward) (Blattner 1999) and ITS4 (reverse) (White et al. 1990) and PCR conditions 94°C/4 min, followed by 30 cycles of 94°C/30 sec, 50°C/1 min, 72°C/1 min and final extension of 72°C/10min. In cases of highly degraded DNA from older herbarium specimens, ITS1 and ITS2 were amplified separately using primers ITS3P (forward) (Möller and Cronk 1997) and the reverse complement ITS2P (reverse). Bidirectional Sanger sequencing was performed by Eurofins (Kentucky, USA) and sequences were checked using Sequencher version 4.8 (Gene Codes).

**Sequence alignment and phylogenetic analysis**

Sequences of 60 individuals were aligned manually using Sequencher and Se-Al (Rambaut 2002). Subunit boundaries follow those determined for *Oryza* (Takaiwa et al. 1985, Yokota et al. 1989) as follows: 18S/ITS1 CATTG/TCGTG; ITS1/5.8S AAATC/CACAC; 5.8S/ITS2 CACGC/CAAAA; ITS2/26S GGACC/GCGAC (an example of a full *Oryza* sequence for location is GenBank accession MF029734). Eight putative hybrids (between the different ribotypes) were excluded from the phylogenetic analysis due to sequence superposition. We included one sample from GenBank (KF753778) as the only previously databased sequence with the ITS-short genotype. Phylogenetic analysis was performed using three approaches: a Neighbour-joining (NJ) analysis with uncorrected (p) distances and 1000 bootstrap replicates, a Maximum Parsimony (MP) analysis with heuristic search (random addition of taxa and TBR branch swapping), both methods being performed in PAUP* (Swofford 2003); and a Maximum Likelihood (ML) analysis using RAxML (v. 8.2.4) with GTRCAT, 1000 rapid bootstraps and Gamma optimisation of tree space run on the CIPRES Science Gateway (Miller et al. 2010, Stamatakis 2014). The MP analysis also included a gap code matrix (for nine gaps: three in *P.arundinacea* and six in outgroup taxa). Sequences are deposited in GenBank under accession numbers: OQ740187-OQ740255.

**Structural analysis of ITS2**

Structural analyses were performed using the ITS2 database (Ankenbrand et al. 2015). We used the *Phalarisarundinacea* ITS2 structure of GenBank accessions FJ821785 (MFE -66.8 kcal/mol) in the ITS2 database for homology modelling (Wolf et al. 2005) of our common variant (ITS-long) as it had a near identical sequence. As homology modelling of the rare variant (ITS-short) fails on FJ821785, alternative templates for homology modelling were investigated. Plausible configurations for ITS2-short were obtained using *Arctagrostislatifolia* (EU792351) and *Phalariscanariensis* (FJ377670) as templates.

**Capillary sizing assay**

A primer was designed using the NCBI Primer-BLAST tool (Ye et al. 2012) ITS2AindelR: 5’-GCAGCCATATCTTCGGC-3’ for use in conjunction with ITS primer ITS3P to allow an accurate sizing assay on an ABI 3730 automated DNA Sequencer (Applied Biosystems). The primer was combined with a M13 tail (5'-TGTAAAACGACGGCCAGT-3') on the forward primer to facilitate fluorescent dye labelling and a further PIG tail (5’-GTTTCTT-3’) on the reverse primer to promote terminal adenylation. We used a hot start touchdown PCR protocol with 95°C/3 min, followed by 10 cycles of 94°C/30 sec, 65°C/30 sec (-1°C per cycle, R 3°C/sec), 72°C/45 sec, followed by a further 30 cycles of 94°C/30 sec, 55°C/30 sec, 72°C/45 sec and a final extension at 72°C/4 min. PCR products were loaded into the capillary machine at 1:30 dilution and traces read using the programme Geneious 8.1.9 (Biomatters Ltd.). The PCR assay was designed to give products of 238 or 245 bp depending on the presence of the 7 bp deletion.

**A sequencing survey and phylogenetic analysis reveals intraspecific divergence in ITS including a 7 bp deletion**

**Figure 2.**
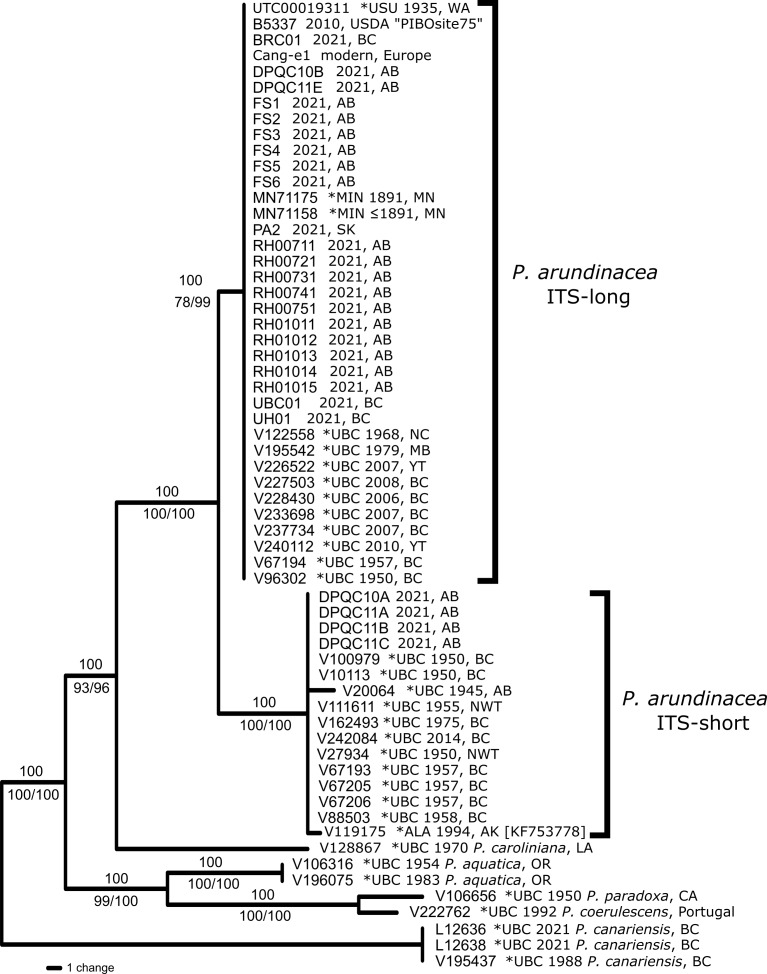
Phylogeny of 53 individuals of *Phalarisarundinacea* and five outgroup taxa, based on ITS variation. Included are 60 individuals sampled for this study as well as the only GenBank sample of *P.arundinacea* found with the ITS-short sequence [KF753778]. Asterisks indicate samples obtained from herbarium material. The tree is a strict consensus from the MP analysis with Majority Rule consensus values above nodes and NJ/ML bootstrap support values below nodes. Two clades can be seen: the deletion clade (ITS-short) and the full length ITS clade (ITS-long). Sample details are given in Tables 1, 2 and Suppl. materials 1, 2, 3.

**Figure 3.**
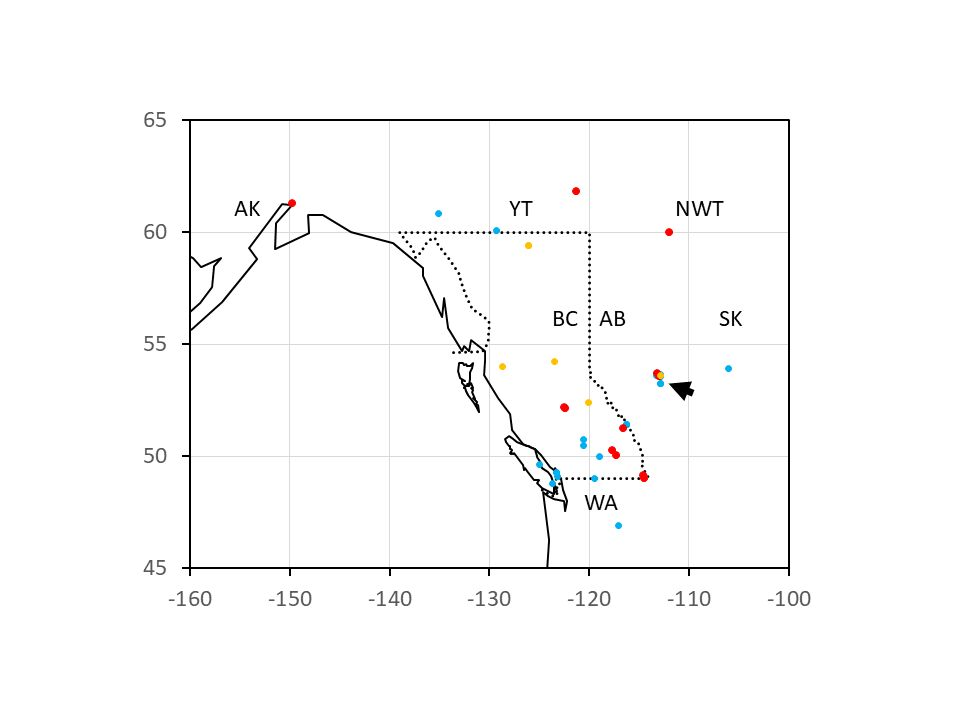
Map of western Canada showing the locations of 51 genotyped samples of Phalarisarundinacea. Dotted line indicates the Province of British Columbia. Red dots show the locations of the “short” ribotypes (n = 13); blue “long” (n = 32) and orange putative hybrids (n = 6). Only three placeholder specimens are given for Elk Island National Park (arrowed; see additional map Suppl. material 4). Sample details are given in Tables 1, 2 and Suppl. materials 1, 2, 3.

Initial results of an ITS sequencing survey of *Phalarisarundinacea* from western Canada revealed two distinctive sequences. One is full length with fixed heterozygosity characteristic of polyploids; the other is shorter, with a 7 bp deletion in ITS2 and with no fixed heterozygous base positions. The differences are summarised in Table 3. The tree topologies recovered from the different phylogenetic approaches were nearly identical. The matrix length was 603 bp (612 characters with gap coding) and the MP search recovered two trees with length 117 (CI: 0.93, RI: 0.98); we present the strict consensus topology in Fig. 2 showing majority rule consensus values as well as NJ and ML bootstrap support values. The best ML model fit for the data (AIC) was GTR+G (-lnL 1611.45). Use of outgroups showed that the full length sequence (which we call ITS-long) was likely the ancestral one and the deletion (ITS-short) is a putatively-derived character so far known only from plants in north-western North America (Fig. 3). When compared with all available world-wide sequences from GenBank (including Asia, Europe, North and South America), only one sequence was found to have the ITS-short genotype (KF753778) from Cook Inlet, Alaska; all other GenBank samples are the ITS-long genotype and ITS-long sequences found in North America are highly similar or identical to European genotypes. Tables 1, 2 show to which clade (ITS-long/-short) historical herbarium specimens can be assigned.

**Figure 4.**
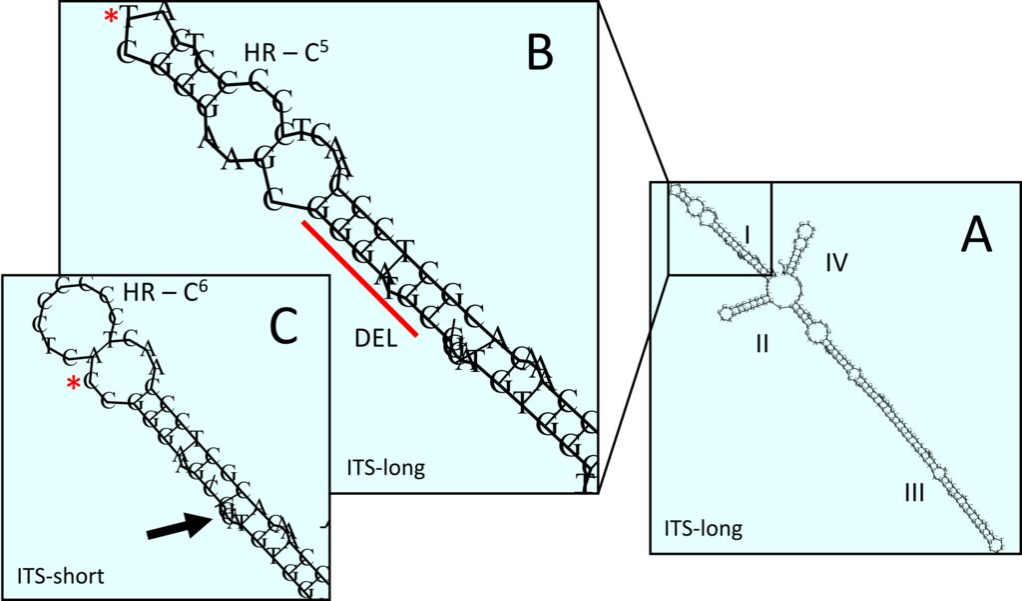
Secondary structure consequences of the deletion in ITS2. A) Predicted secondary structure of *Phalarisarundinacea* ITS2, based on the common variant (ITS2-long). B) Detail of helix I; DEL = the bases (GGGATGC) deleted in the ITS2-short variant; HR – C5 = 5 cytosine homopolymer repeat; asterisk T – position of the T/C single nucleotide polymorphism (aligned position 413). C) Possible alternative structure of helix I in the ITS2-short variant, based on homology modelling using Arctagrostislatifolia as the template; the cytosine homopolymer repeat is now C6 (6 cytosines); the arrow shows the position of the deleted sequence.

The 7 bp deletion alters the secondary structure of helix I of ITS2. The predicted secondary structure of the common variant (ITS2-long) of *Phalarisarundinacea* ITS2 is the usual eukaryotic four helix model (Fig. 4). Homology modelling of the structure of the ITS2-short sequence against this structure fails, as helix I, which has the 7 bp deletion, is not a suitable model. However, homology modelling with a related grass of similar ITS2 sequence suggests a plausible model for helix I despite the deletion (Fig. 4).

**A PCR-based capillary sizing assay allows rapid detection of the 7 bp deletion clade**

In order to genotype individuals without sequencing, we developed a primer that amplifies a 238 vs. 245 bp amplicon (short enough to size accurately to a single base pair on a capillary machine). ITS-long gave a clear peak at 245 bp and a complete absence of a peak at 238 bp. Despite using a design to promote terminal adenylation (see Methods), if there is a large amount of starting DNA, this peak was split, showing a peak or shoulder at 244 bp. However, in all cases, the fully adenylated peak was unambiguous and as strong or stronger than the unadenylated peak. ITS-short samples gave a strong, unambiguous peak at 238 bp. Product without terminal adenylation sometimes showed as a shoulder, but never a separate peak. ITS-short samples sometimes showed a small peak at 245 bp, but the 238 peak was, in all cases, much stronger. A total of 68 individuals were sized with this method, providing clade (ITS-long/-short/hybrid) affiliation for an additional 34 individuals. Putative hybrids (10 individuals) were identified either by both sequencing and sizing assay (seven individuals), sequence data only (one individual) and two specimens identified as ITS-short in the length assay, but were determined as a putative hybrid with sequence data (Suppl. material 1).

**A survey of Elk Island National Park, Alberta, reveals presence of both ITS ribotypes**

Using the molecular tools detailed above, we were able to conduct extensive sampling of Elk Island National Park (EINP), Alberta. *Phalarisarundinacea* is extremely abundant at EINP and the material in the Park tends to be strongly spreading-rhizomatous and invasive. EINP is bisected into a northern and southern portion by the east-west highway 16. These portions have different management histories, with the northern portion experiencing much greater public access and road development. We refer to these portions as north EINP and south EINP. In all sampled localities of north EINP, ITS-long was the only genotype detected (except a few possible hybrids at Tawayik Lake). In south EINP the situation is very different. Of the 12 individuals genotyped from south EINP, five were ITS-short (DPQC10A and DPQC11A-D).

## Geographic coverage

### Description

North-western North America

## Taxonomic coverage

### Description

*Phalarisarundinacea*, *P.aquatica*, *P.canariensis*, *P.caroliniana*, *P.coerulescens* and *P.paradoxa*.

## Usage licence

### Usage licence

Creative Commons Public Domain Waiver (CC-Zero)

## Data resources

### Data package title

Specimen details for all 94 samples genotyped (86 *Phalarisarundinacea* and eight outgroup taxa sampled).

### Number of data sets

1

### Data set 1.

#### Data set name

Specimen details for all 94 samples genotyped (86 *Phalarisarundinacea* and eight outgroup taxa sampled).

#### Description

Suppl. material 3 contains specimen details for all 94 samples genotyped (86 *Phalarisarundinacea* and eight outgroup taxa sampled).

**Data set 1.** 

Column label	Column description
occurrenceID	Specimen Code identifier for the Occurrence.
basisOfRecord	Specimen type as the specific nature of the data record.
eventDate	Date of specimen collection.
eventRemarks	Note of incomplete date information.
decimalLatitude	The geographic latitude (in decimal degrees, using the spatial reference system given in geodeticDatum) of the geographic centre of a Location.
decimalLongitude	The geographic longitude (in decimal degrees, using the spatial reference system given in geodeticDatum) of the geographic centre of a Location.
geodeticDatum	The ellipsoid, geodetic datum or spatial reference system (SRS) upon which the geographic coordinates given in decimalLatitude and decimalLongitude are based.
eventRemarks	Ribotype of ITS sequence.
country	The name of the country or major administrative unit in which the Location occurs.
locality	The specific description of the place.
verbatimLocality	The original textual description of the place.
scientificName	The full scientific name, with authorship.
identificationQualifier	Qualifier on current identification.
taxonRank	The taxonomic rank of the most specific name in the scientificName.
institutionCode	The name (or acronym) in use by the Herbarium institution having custody of the object(s) or information referred to in the record.
collectionCode	The name, acronym, coden or initialism identifying the collection or dataset from which the record was derived.

## Additional information

**Implications of two highly divergent intraspecific ribotypes**

The making of a ribosome is a complex process: it involves multiple steps and over 200 biogenesis factors (Sáez-Vásquez and Delseny 2019). In this process, ITS2 plays an important role. The excision of ITS2 from the pre-ribosomal RNA is essential to generate mature 25/26S and 5.8S and the secondary structure of ITS2 is important for this process (Schultz et al. 2005). Embryophytes have four helices (numbered I-IV) arising from a central ring. These helices require complementary base pairing to form (and be stable). They are, therefore, generally quite conserved in sequence, with mutations only surviving if they preserve the pairing energetics of the helix (Zhang et al. 2020). For this reason, it is surprising to see an intraspecific seven base-pair deletion in helix I. In addition, this helix carries an SNP and an extra cytosine in a cytosine repeat sequence. There is an energetically plausible alternative structure for this helix, but it still represents a marked change in helix pairing structure. Furthermore, there are seven SNPs in helix III (although these do not markedly impact helix structure). Given this, it is evident that there are two distinctive ITS2 ribotypes in north-western Canada, being distinguished by two indel events, one with a major impact on helix nucleotide pairing and five SNPs.

The ITS-long sequence was highly similar or identical to sequences of known European genotypes obtained from GenBank. In contrast, the ITS-short individuals are often from non-agricultural and remote localities, (e.g. Yoho NP and Cook Inlet Lowlands of Alaska and North West Territory) or from older herbarium specimens (e.g. a 1945 specimen from Fort Saskatchewan, AB). These ITS-short genotypes are almost uniformly from riparian and lacustrine habitats and never grassland. This genotype is currently unknown outside north-western North America. Of historical and previously studied samples, the late 19^th^ century samples (1891), obtained from mid-western North America, Minnesota and proposed as native genotypes in that region by Noyszewski et al. (2021), had the ITS-long genotype in our study; a 1935 specimen from Pullman, Washington, proposed to be an early European introduction by Kettenring et al. (2019), also had the ITS-long genotype; and a modern (2010) specimen from remote northern BC (Kitimat), interpreted as native by Jakubowski et al. (2014), but of mixed heritage by Kettenring et al. (2019), also had ITS-long in our study. In summary therefore, across North America, the ITS-long genotype may be present in both native and introduced RCG, whereas the ITS-short genotype appears to be a localised variant in the Pacific northwest.

The existence of distinctive North American genotype(s) (e.g. Noyszewski et al. (2021)) suggests that RCG was widespread in North America prior to the massive seeding of introduced agronomic genotypes in forage and revegetation seed mixes (Merigliano and Lesica 1998). However, there is still much uncertainty and, in some cases, disagreement, regarding the extent of distribution and location of present-day native RCG populations in North America (Jakubowski et al. 2013). One potential use of our relatively easily scored genetic marker would be to establish representation and association of the different ribotypes in native populations. Preliminary observations of the growth forms of our sampled RCG suggests that specimens with the ITS-short ribotype tended to be smaller, less strongly rhizomatous and were not noted to be invasive. However, there is no morphologically reliable method of distinguishing native from invasive RCG (Kettenring et al. 2019). The main indicators are vigour of growth and rhizomatous spread, but the usefulness of these indicators is uncertain and subject to environmental variation. Presently, molecular markers will likely remain the primary means of making broad surveys of RCG, to determine the geographical and ecological patterns of native persistence and to identify cryptic invasions of RCG across North America and the potential signature of intraspecific hybrids.

## Supplementary Material

909AC0EB-3121-5EAA-B684-34C4376DFE9C 10.3897/BDJ.11.e101257.suppl1 Supplementary material 1 Ten putative hybrids between ITS-long and ITS-short clades. Data type occurrences Brief description Ten putative hybrids between ITS-long and ITS-short clades. Seven based on both sequence and assay data, one based on sequence data only (marked ^) and two samples which appeared hybrid in sequence data, but ITS-short in sizing assay data (marked with *). Region abbreviations: AB Alberta, BC British Columbia. File: oo_787189.docx https://binary.pensoft.net/file/787189 Diana M. Percy, Quentin C. B. Cronk

3CBAB3D5-AD6D-58EA-813F-F2A2285284E6 10.3897/BDJ.11.e101257.suppl2 Supplementary material 2 Herbarium specimens used as outgroups. Data type occurrences Brief description Herbarium specimens used as outgroups. File: oo_787190.docx https://binary.pensoft.net/file/787190 Diana M. Percy, Quentin C. B. Cronk

699D0AF6-A5A4-5F14-A1E5-60BE1CE6A3BF 10.3897/BDJ.11.e101257.suppl3 Supplementary material 3 Specimen details for all 94 samples genotyped. Data type occurrences Brief description Specimen details for all 94 samples genotyped (86 Phalarisarundinacea and eight outgroup taxa sampled). File: oo_802456.csv https://binary.pensoft.net/file/802456 Diana M. Percy, Quentin C. B. Cronk

D309CCD8-0A6C-5079-B04B-93DD6C5AF107 10.3897/BDJ.11.e101257.suppl4 Supplementary material 4 Map of Elk Island National Park with the locations of 38 genotyped samples marked. Data type occurrences Brief description Map of Elk Island National Park with the locations of 38 genotyped samples marked. Red crosses show the locations of the “short” ribotypes (n = 4); blue crosses “long” (n = 29), and orange circles putative hybrids (n = 5). File: oo_787193.docx https://binary.pensoft.net/file/787193 Diana M. Percy, Quentin C. B. Cronk

## Figures and Tables

**Table 1.** Herbarium specimens identified as ITS-short: determined by sequencing or sizing assay to have a 7 bp deletion in ITS2. An asterisk indicates one individual identified as ITS-short in assay data, but putative hybrid in sequence data; and [] indicates the only sequence found on GenBank with the ITS-short genotype. Region abbreviations: AB Alberta, AK Alaska, BC British Columbia, NWT Northwest Territories.

**Accession no.**	**Herb.**	**Date**	**Locality**	**Region**	**Habitat**
V100979	UBC	1950	Chilcotin, Madden Lake	BC	not recorded
V101113	UBC	1950	Chilcotin, Meldrum Creek	BC	marsh
*V152455	UBC	1974	near Shamrock, ca. 30 miles northwest of Prince George	BC	in post-glacial bed of the Stuart River
V162493	UBC	1975	Beaver Lake, Wilson Creek Road, nr. Slocan Lake	BC	swampy lake edge
V27934	UBC	1950	S. of Ft Smith	NWT	scattered in clumps along dried-up slough
V67193	UBC	1957	Kootenay District, Flathead, Procter Lake	BC	in 2 ft (60 cm) of water at lake edge
V67205	UBC	1957	Kootenay District, Sage Creek Lodge.	BC	wet edge of slough
V67206	UBC	1957	Flathead valley, Marl Lake	BC	wet edge of lake
V88503	UBC	1958	Kootenay, Nakusp, Wilson Lake.	BC	in peat bog
V111611	UBC	1955	5 mi (7.5 km) southeast of Fort Simpson,	NWT	rare in moist black ground in Carex meadow
V242084	UBC	2014	Yoho National Park, Hoodoo Creek Campground area	BC	somewhat calcareous swampy lakeshore
V119175 [KF753778]	ALA	1994	Cook Inlet lowlands, Otter Creek at Loop Road	AK	herbaceous border of ponded creek
V20064	UBC	1945	Just east of Fort Saskatchewan	AB	creek bottom

**Table 2.** Herbarium specimens identified as ITS-long: determined by sequencing or sizing assay to lack the 7 bp deletion in ITS2. Region abbreviations: BC British Columbia, MB Manitoba, MN Minnesota, NC North Carolina, WA Washington, YT Yukon Territory.

**Accession no.**	**Herb.**	**Date**	**Locality**	**Region**	**Habitat**
V226522	UBC	2007	Alaska Highway km 1016	YT	apparently seeded along highway
V67194	UBC	1957	Sage Creek, Flathead	BC	grassy meadow
V96302	UBC	1950	Salmon Arm	BC	not recorded
V97215	UBC	1962	Thompson-Nicola Regional District, Tranquille	BC	wet meadow
V233698	UBC	2007	Greater Vancouver, Delta, Westham Island	BC	tidal shore (var. *picta*)
V122558	UBC	1968	Avery County, Elk River at Heaton	NC	marsh
V228430	UBC	2006	Osoyoos, Haynes Point Provincial Park	BC	meadow beside wetland
V195542	UBC	1979	Pencil Lake, Riding Mountain National Park	MB	road allowance, jet ski trail
V240112	UBC	2010	Whitehorse	YT	sewage treatment facility
V227503	UBC	2008	Vancouver Island, Duncan, Somenos Marsh	BC	thick grassy marsh margin
V237734	UBC	2007	Vancouver Island, Cumberland	BC	roadside with introduced grasses
UTC00019311	USU	1935	Palouse River, Pullman	WA	shallow pools of drying streambed
MN71158	MIN	≤1891	St Anthony Park, Ramsey	MN	see Noyszewski et al. (2021)
MN71175	MIN	1891	Ramsey	MN	see Noyszewski et al. (2021)

**Table 3.** Molecular characteristics of the 7 bp deletion clade (ITS-short) in comparison to the full length variant (ITS-long). Length variation in *Phalarisarundinacea* is caused by one 7 bp deletion and a 1 bp homopolymer indel, giving a combined length difference of 6 bp. The aligned sequence length for 52 *Phalarisarundinacea* individuals using the ITS1-5.8S-ITS2 subunit boundaries following Takaiwa et al. (1985) and Yokota et al. (1989) is 600 bp and, including six outgroup taxa (eight individuals), it is 603 bp. Ambiguity codes (Y, R, S) are given for heterozygotes. Sites homozygous, but polymorphic between different individuals, are given as C/T etc. Individuals that were interpreted as putative hybrids are given in Suppl. material 1.

**Feature**	**ITS-long**	**ITS-short**	**Outgroups**
No. of individuals	37	15	8
Total sequence length, ITS1-5.8S-ITS2 (bp)	599 (no variation)	593 (no variation)	598-600
ITS1 length	219	219	219-222
5.8S length	164	164	164
ITS2 length	216	210	213-216
Fixed heterozygosity in ITS1-long (aligned position)	Y(30), Y(181), Y(193), S(208)	C(30), C(181), C(193), C(208)	T/C(30), C(181), T/C(193), C(208)
Fixed heterozygosity in 5.8S-long (aligned position)	Y(345), Y(359)	C(345), C(359)	C(345), C/T(359)
Fixed heterozygosity in ITS2-long (aligned position)	Y(421), R(489), Y(587)	C(421), A/R(489), C(587)	C/T(421), G(489), C(587)
Fixed SNPs between groups in ITS1 (aligned position)	A(7), C(60), C(195)	C(7), T(60), Y/T(195)	C(7), C/T(60), C(195)
Fixed SNPs between groups in ITS2 (aligned position)	T(413), C(493), T(528)	C(413), T/Y(493),C/Y(528)	T(413), C(493), T(528)
SSR in ITS2 (aligned position)	C^5^(404-408)	C^6^(404-409)	C^2-6^(404-409)
