# Supplementary material for: Report of two distinct ribotypes in ITS sequences of Phalarisarundinacea (Poaceae) in western Canada and Alaska
Source: Biodivers Data J. 2023 Apr 11;11:e101257. doi: 10.3897/BDJ.11.e101257 (PMC10848705; doi:10.3897/BDJ.11.e101257)
Supplement: Supplementary material 1 — Ten putative hybrids between ITS-long and ITS-short clades. [file bdj-11-e101257-s001.docx]

**Report of two distinct ribotypes in ITS sequences of *Phalaris arundinacea* (Poaceae) in western Canada and Alaska**

Diana M. Percy^1*^, Quentin C. B. Cronk^1,2^

^1^ *Department of Botany and Biodiversity Research Centre, University of British Columbia, Vancouver, BC, Canada*

^2^ *Beaty Biodiversity Museum, University of British Columbia, Vancouver, BC, Canada*

^*^**Corresponding author:** Diana M. Percy (email: [diana.percy@ubc.ca](mailto:diana.percy@ubc.ca))

Supplementary Table 1. Ten putative hybrids between ITS-long and ITS-short clades. Seven based on both sequence and assay data, one based on sequence data only (marked ^), and two samples which appeared hybrid in sequence data but ITS-short in sizing assay data (marked with *). Region abbreviations: AB Alberta, BC British Columbia.

| **Accession no.** | **Herb.** | **Date** | **Locality** | **Region** | **Habitat** |
| --- | --- | --- | --- | --- | --- |
| DPQC7C | UBC | 2021 | Elk Island National Park, Tawayik Lake | AB | lake shore with extensive grasslands |
| DPQC8B | UBC | 2021 | Elk Island National Park, Tawayik Lake | AB | lake shore with extensive grasslands |
| DPQC8C | UBC | 2021 | Elk Island National Park, Tawayik Lake | AB | lake shore with extensive grasslands |
| DPQC8D | UBC | 2021 | Elk Island National Park, Tawayik Lake | AB | lake shore with extensive grasslands |
| V196810 | UBC | 1960 | Liard Hot Springs | BC | clearing near hot pool |
| V121059 | UBC | 1943 | Liard Hot Springs | BC | not recorded |
| V170917 | UBC | 1980 | Azure Lake, Wells Gray Park | BC | lakeshore |
| ^Cang-n7 | n/a | 1973 | Kitimat | BC | “USA Jakubowski” USDA (PI 387929), see Kettenring et al. 2019 |
| *DPQC11D | UBC | 2021 | Elk Island National Park (southen part), Robs Road | AB | pond margin |
| *V152455 | UBC | 1974 | near Shamrock, ca 30 miles northwest of Prince George | BC | in post-glacial bed of the Stuart River |
