# Supplementary material for: Report of two distinct ribotypes in ITS sequences of Phalarisarundinacea (Poaceae) in western Canada and Alaska
Source: Biodivers Data J. 2023 Apr 11;11:e101257. doi: 10.3897/BDJ.11.e101257 (PMC10848705; doi:10.3897/BDJ.11.e101257)
Supplement: Supplementary material 2 — Herbarium specimens used as outgroups. [file bdj-11-e101257-s002.docx]

**Report of two distinct ribotypes in ITS sequences of *Phalaris arundinacea* (Poaceae) in western Canada and Alaska**

Diana M. Percy^1*^, Quentin C. B. Cronk^1,2^

^1^ *Department of Botany and Biodiversity Research Centre, University of British Columbia, Vancouver, BC, Canada*

^2^ *Beaty Biodiversity Museum, University of British Columbia, Vancouver, BC, Canada*

^*^**Corresponding author:** Diana M. Percy (email: [diana.percy@ubc.ca](mailto:diana.percy@ubc.ca))

Supplementary Table 2. Herbarium specimens used as outgroups.

| **Accession no.** | **Herb.** | **Date** | **Species** | **Region of origin** |
| --- | --- | --- | --- | --- |
| V106316 | UBC | 1954 | *Phalaris aquatica* | Oregon |
| V196075 | UBC | 1983 | *Phalaris aquatica* | Oregon |
| L12636 | UBC | 2021 | *Phalaris canariensis* | British Columbia |
| L12638 | UBC | 2021 | *Phalaris canariensis* | British Columbia |
| V195437 | UBC | 1988 | *Phalaris canariensis* | British Columbia |
| V128867 | UBC | 1970 | *Phalaris caroliniana* | Louisiana |
| V222762 | UBC | 1992 | *Phalaris coerulescens* | Portugal |
| V106656 | UBC | 1950 | *Phalaris paradoxa* | California |
