# Supplementary material for: Report of two distinct ribotypes in ITS sequences of Phalarisarundinacea (Poaceae) in western Canada and Alaska
Source: Biodivers Data J. 2023 Apr 11;11:e101257. doi: 10.3897/BDJ.11.e101257 (PMC10848705; doi:10.3897/BDJ.11.e101257)

**Report of two distinct ribotypes in ITS sequences of *Phalaris arundinacea* (Poaceae) in western Canada and Alaska**

Diana M. Percy^1*^, Quentin C. B. Cronk^1,2^

^1^ *Department of Botany and Biodiversity Research Centre, University of British Columbia, Vancouver, BC, Canada*

^2^ *Beaty Biodiversity Museum, University of British Columbia, Vancouver, BC, Canada*

^*^**Corresponding author:** Diana M. Percy (email: [diana.percy@ubc.ca](mailto:diana.percy@ubc.ca))

Supplementary Figure 1. Map of Elk Island National Park with the locations of 38 genotyped samples marked. Red crosses show the locations of the “short” ribotypes (n=4); blue crosses “long” (n=29), and orange circles putative hybrids (n=5).


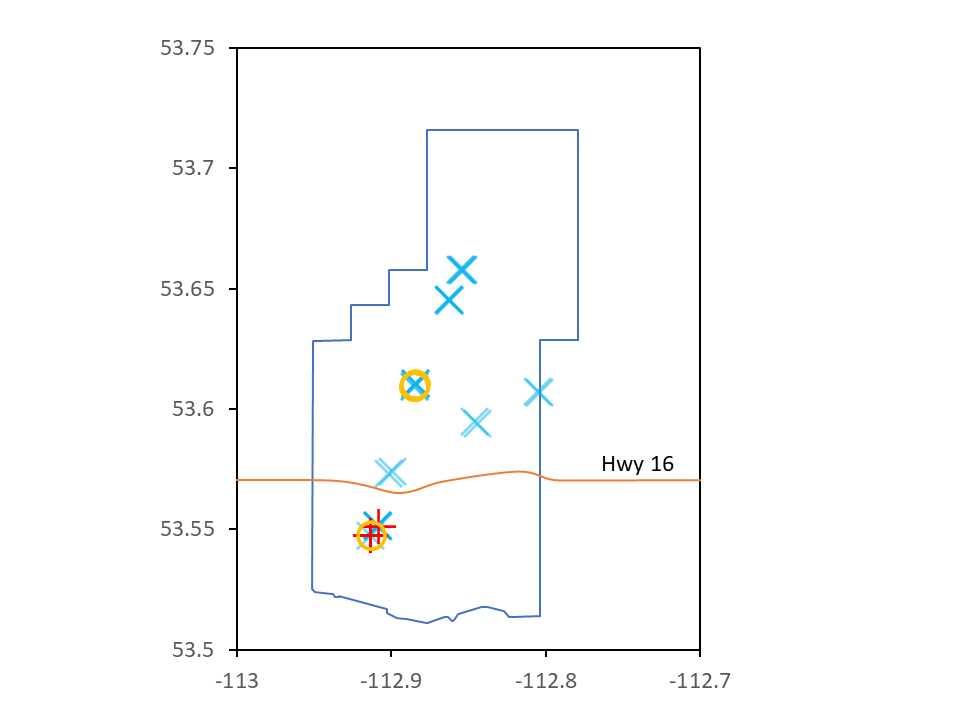

Supplement: Supplementary material 4 — Map of Elk Island National Park with the locations of 38 genotyped samples marked. [file bdj-11-e101257-s004.docx]
